# Interleukin-33 induces interleukin-8 expression via JNK/c-Jun/AP-1 pathway in human umbilical vein endothelial cells

**DOI:** 10.1371/journal.pone.0191659

**Published:** 2018-01-26

**Authors:** Katsuyuki Umebashi, Akinori Tokito, Masayoshi Yamamoto, Michihisa Jougasaki

**Affiliations:** 1 Institute for Clinical Research, National Hospital Organization Kagoshima Medical Center, Kagoshima, Japan; 2 Neurohumoral Biology, Cooperative Department of Innovative Medicine, Kagoshima University Graduate School of Medical and Dental Sciences, Kagoshima, Japan; Medical College of Wisconsin, UNITED STATES

## Abstract

Interleukin (IL)-33 is a member of the IL-1 cytokine family with dual functions as a traditional cytokine and as a transcriptional regulator. We recently reported that IL-33 up-regulated growth regulated oncogene (GRO)-α/CXCL1 expression in human vascular endothelial cells. The aim of this study was to investigate the effect of IL-33 on the expression of IL-8/CXCL8, another member of the CXC-chemokine family, and to elucidate its signaling pathways in human umbilical vein endothelial cells (HUVECs). Immunocytochemical staining and Western immunoblot analysis revealed that IL-33 augmented IL-8 protein expression in HUVECs. Real time reverse transcription-polymerase chain reaction (RT-PCR) and enzyme-linked immunosorbent assay (ELISA) showed that IL-33 significantly increased IL-8 mRNA and secretion in a dose- and time-dependent manner. IL-33 preferentially stimulated proliferating subconfluent cells, and increased IL-8 secretion to a higher level compared with confluent cells. IL-33 also stimulated phosphorylations of c-Jun N-terminal kinase (JNK) and c-Jun, and enhanced activator protein (AP)-1 DNA-binding activity, all of which were suppressed by SP600125, a JNK inhibitor. Moreover, IL-33-induced IL-8 mRNA and secretion were also suppressed by SP600125. Transfection of c-Jun small interfering RNA into cultured HUVECs significantly reduced the IL-33-induced increase in IL-8 secretion from HUVECs. The present study demonstrates that IL-33 induces IL-8 expression via JNK/c-Jun/AP-1 pathway in human vascular endothelial cells, and provides a new insight into the role of IL-33-induced IL-8 in the pathophysiology of atherosclerosis and vascular inflammation.

## Introduction

Although atherosclerosis is considered a chronic inflammatory disease that causes acute cardiovascular events [1, 2], the regulation of inflammation in atherosclerosis is not fully clarified yet. Attachment of the leukocytes to the endothelial cells and subsequent migration of leukocytes into the subendothelial space are the initial events in atherosclerosis, and chemokines play an important role in its pathogenesis. Chemokines are small molecule inflammatory proteins and are divided into four canonical subclasses according to the position of the N-terminal cysteine residues: C, CC, CXC, and CX3C chemokines [3]. Interleukin (IL)-8, also named CXCL8, belongs to the CXC-chemokine family together with growth regulated oncogene (GRO)-α/CXCL1. IL-8 is present in elevated levels in human coronary atherosclerotic tissues [4], and macrophages are considered the major site of IL-8 production in atherosclerotic plaque tissues [5]. Recombinant human IL-8 induced endothelial cell proliferation and capillary tube formation in human vascular endothelial cells, suggesting that IL-8 is also associated with angiogenesis [6]. Circulating levels of IL-8 significantly increased in patients with unstable angina pectoris compared with normal control subjects [7, 8]. In addition, elevated levels of plasma IL-8 were associated with the increased risk of future coronary arterial diseases in healthy subjects [9]. These findings suggest that IL-8 plays an important role in the pathogenesis of atherosclerosis.

IL-33, originally identified as a nuclear factor in post-capillary high endothelial venules, is a proinflammatory cytokine that belongs to the IL-1 cytokine family [10]. IL-33 is thereafter found to be a ligand for the orphan receptor, suppression of tumorigenecity (ST) 2 [11, 12]. As a cytokine, IL-33 is released from various cells in response to tissue damage or mechanical strain to alert the immune system, and acts as an “alarmin” [13, 14]. IL-33 binds to its transmembrane receptor ST2 on the inflammatory cells, and induces T helper type 2 lymphocytes (Th2)-associated cytokines and chemokines [15, 16]. The signal transduction system induced by IL-33 includes activation of mitogen activated protein kinase (MAPK), such as c-Jun N-terminal kinase (JNK), p38 MAPK, and extracellular signal-regulated kinase (ERK) 1/2 [16, 17]. However, the role of IL-33 in the pathophysiology of atherosclerosis is not fully understood yet. Demyanets et al. demonstrated that IL-33 up-regulated adhesion molecules and chemokines, such as vascular cell adhesion molecule (VCAM)-1, intercellular adhesion molecule (ICAM)-1, E-selectin, and monocyte chemoattractant protein (MCP)-1/CCL2 in the endothelial cells [18]. Other investigators reported that plasma levels of IL-33 were significantly higher in patients with acute coronary syndrome compared with stable angina or control subjects [19], and that plasma IL-33 levels predicted carotid plaque progression in patients with early rheumatoid arthritis [20], raising the possibility that IL-33 is involved in the pathophysiology of atherosclerosis. Accumulating evidence has revealed that IL-33 induces IL-8, one of the CXC-chemokines, in various types of cells, such as goblet cells, normal human bronchial epithelial cells, and human vascular endothelial cells [21–24]. More recently, we have demonstrated that IL-33 increases expression of GRO-α, another member of the CXC-chemokine family, via activating MAPK and nuclear factor (NF)-κB in human umbilical vein endothelial cells (HUVECs) [25]. Therefore, the present study was designed to investigate whether IL-33 influences IL-8 expression in HUVECs, and to explore the underlying signaling pathways, especially JNK pathway in these cells.

## Materials and methods

### Reagent

Recombinant human IL-33 and recombinant human IL-1β were purchased from PeproTech (Rocky Hill, NJ). The mouse monoclonal anti-human IL-8 antibody, recombinant human ST2/IL-33R Fc Chimera, and the mouse IgG isotype control were from R&D System (Minneapolis, MN). The rabbit polyclonal antibodies specific for JNK, phospho-JNK (Thr183/Tyr185), c-Jun, and phospho-c-Jun (Ser63) were obtained from Cell Signaling Technology (Beverly, MA). SP600125 was purchased from BIOMOL (Plymouth Meeting, PA).

### Cell culture of HUVECs

HUVECs were purchased from Kurabo (Osaka, Japan), and seeded in plastic plates precoated with type I collagen (Asahi Techno Glass, Nagoya, Japan). The cells were maintained in medium 199 (Life Technologies, Carlsbad, CA) supplemented with 10% heat-inactivated fetal calf serum (FCS), 0.5 μg/mL fungizone, 0.25 μg/mL amphotericin B, 100 mg/mL streptomycin, 100 U/mL penicillin (Life Technologies, Carlsbad, CA), 14 U/mL heparin (Ajinomoto, Tokyo, Japan), 20 μg/mL endothelial cell growth supplement (Kohjin Bio, Saitama, Japan), and 10 μg/mL human epidermal growth factor (PeproTech, Rocky Hill, NJ). HUVECs were cultured at 37°C in 5% CO_2_ and 95% air in a humidified atmosphere. At confluence, HUVECs formed a typical “cobblestone” monolayer. HUVECs at third to seventh passage were used in the present experiments. After serum starvation with medium 199 supplemented with 0.5% FCS, the cell culture experiments were performed.

### Immunocytochemistry

HUVECs plated on a Biocoat slide glass (BD Biosciences, San Jose, CA) were fixed with 1% buffered paraformaldehyde (Wako Pure Chemical, Osaka, Japan) for 20 min, and used for the immunocytochemical staining. The indirect immunoperoxidase method was used for the immunocytochemical analysis, as described previously [26]. The primary antibody against IL-8 was used at the concentration of 20μg/mL. The specificity of the immunostaining was confirmed by substitution of the normal mouse IgG for the primary antibody.

### Western immunoblot analysis

Western immunoblot analysis was examined as previously described with a few modifications [26, 27]. Briefly, HUVECs were washed with cold PBS and immediately harvested in ice-cold cell lysis buffer together with phenylmethylsulfonyl fluoride and protease inhibitor cocktail. Aliquots of proteins were resuspended in sodium dodecyl sulfate sample buffer and dithiothreitol, sonicated, boiled for 5 min, and separated by 4–12% NuPAGE Bis-Tris gels (Life Technologies, Carlsbad, CA). The proteins were transferred to a polyvinylidene difluoride membrane by electroblotting for 2 hours. The membrane was soaked in 5% nonfat dry milk blocking buffer, and then incubated with the primary antibodies overnight at 4°C at concentrations as suggested by the manufacture. After washing, the membrane was incubated with horseradish peroxidase-conjugated secondary antibody (Cell Signaling Technology, Beverly, MA) for 1 hr. The protein bands were visualized by ECL prime (GE Healthcare, Buckinghamshire, UK). The intensities of the blots were analyzed using a ChemiDoc Touch Imaging System (Bio-Rad, Hercules, CA).

### Total RNA extraction and real time reverse transcription-polymerase chain reaction (RT-PCR)

Total RNA was extracted from HUVECs using Pure Link RNA Mini kit (Invitrogen, Carlsbad, CA). cDNA was synthesized with Superscript Reverse Transcriptase (Invitrogen, Carlsbad, CA). Real time PCR was performed using Power SYBR Green PCR Master Mix (Applied Biosystems, Warrington, UK) on a CFX connect thermal cycler (Bio-Rad, Hercules, CA). The value of each cDNA was calculated using the ΔΔCq method and normalized to the value of the housekeeping gene, glycerol-3-phosphate dehydrogenase (GAPDH).

Oligonucleotide PCR primers targeting IL-8 mRNA was designed according to a previous report [28], and the specificity of the primers was confirmed by BLAST search and melting curve analysis. The primer sequences were as follows:

IL-8 forward: 5’-AAGAAACCACCGGAAGGAAC-3’

IL-8 reverse: 5’-ACTCCTTGGCAAAACTGCAC-3’

GAPDH forward: 5’-GCACCGTCAAGGCTGAGAAC-3’

GAPDH reverse: 5’-TGGTGAAGACGCCAGTGGA-3’

### Enzyme-linked immunosorbent assay (ELISA)

Concentrations of IL-8 in the culture medium were determined by using a human IL-8 ELISA kit (R&D Systems, Minneapolis, MN) according to the manufacture’s protocol. Briefly, after incubation, the culture medium was removed. Samples and standards were added to each well of microplate, which was precoated with anti-human IL-8 monoclonal antibody, and incubated for 2 hours. Each well was washed and incubated with the enzyme-linked polyclonal antibody specific for human IL-8 for 2 hours. The wells were washed to remove unbound antibody-enzyme reagent, and substrate solution was added to each well. After incubation for 20 min at room temperature, the enzyme reaction was stopped. IL-8 concentrations were determined by comparison of the optical density results with the standard curve.

### Immunofluorescence staining

HUVECs plated on a BioCoat slide glass (BD biosciences, San Jose, CA) were treated with IL-33 in the presence or absence of SP600125 for 60 min. After incubation, the cells were fixed with 4% paraformaldehyde and permeabilized with 0.1% Triton X-100. The slides were blocked with normal horse serum for 30 min, and then incubated with the rabbit phospho-c-Jun antibody at 800-fold dilution. After overnight incubation, the slides were washed and incubated with anti-rabbit IgG-Alexa (Cell Signaling Technology, Beverly, MA) at 250-fold dilution for 1 hour, and counterstained the nuclei with Hoechst 33342 (Invitrogen, Carlsbad, CA) for 5 min. Stained slides were analyzed by fluorescence microscopy (Olympus, Tokyo, Japan).

### Preparation of nuclear extracts and electrophoretic mobility shift assay (EMSA)

EMSA was performed using an EMSA gel-shift kit (Panomics, Santa Clara, CA) as previously described with some modifications [26]. Briefly, after HUVECs were washed with cold PBS and scraped, the cell pellets were collected by centrifugation. The NucBuster protein extraction kit (Novagen, Madison, WI) was used to extract nuclear protein from HUVECs. Nuclear extracts were incubated with the biotin-labeled activator protein (AP)-1 consensus probe (5’-CGCTTGATGACTCAGCCGGAA-3’) at room temperature for 30 min. The protein-DNA complexes were separated by electrophoresis on a 6% DNA retardation gel (Invitrogen, Carlsbad, CA), and electronically transferred to nylon membrane. For chemiluminescence band detection, the membrane was incubated with horseradish peroxidase-streptavidin solution and chemiluminescence reagent. The intensities of the blots were analyzed using a ChemiDoc Touch Imaging System (Bio-Rad, Hercules, CA).

### Transfection with small interfering RNA (siRNA)

Transfection with siRNA was performed according to the manufacture’s protocol (Invitrogen, Carlsbad, CA) with some modifications [29]. Transfection complexes were prepared using siRNA reagent, transfection medium, and c-Jun siRNA, and were delivered to cell monolayers with 100 nmol/L final concentration of siRNA duplexes. A control scrambled siRNA was used as a negative control.

### Statistical analysis

Results of quantitative studies are expressed as means ± SEM. Each data point represents the average of three to six independent experiments. Student’s t-test was used for Western immunoblot analysis. One-way ANOVA test was used to make comparisons among three or more groups and the Tukey-Kramer’s post-hoc test was used to identify differences between two groups. P value <0.05 were considered statistically significant.

## Results

### Immunocytochemical staining and Western immunoblot analysis for IL-8

**Fig 1:**
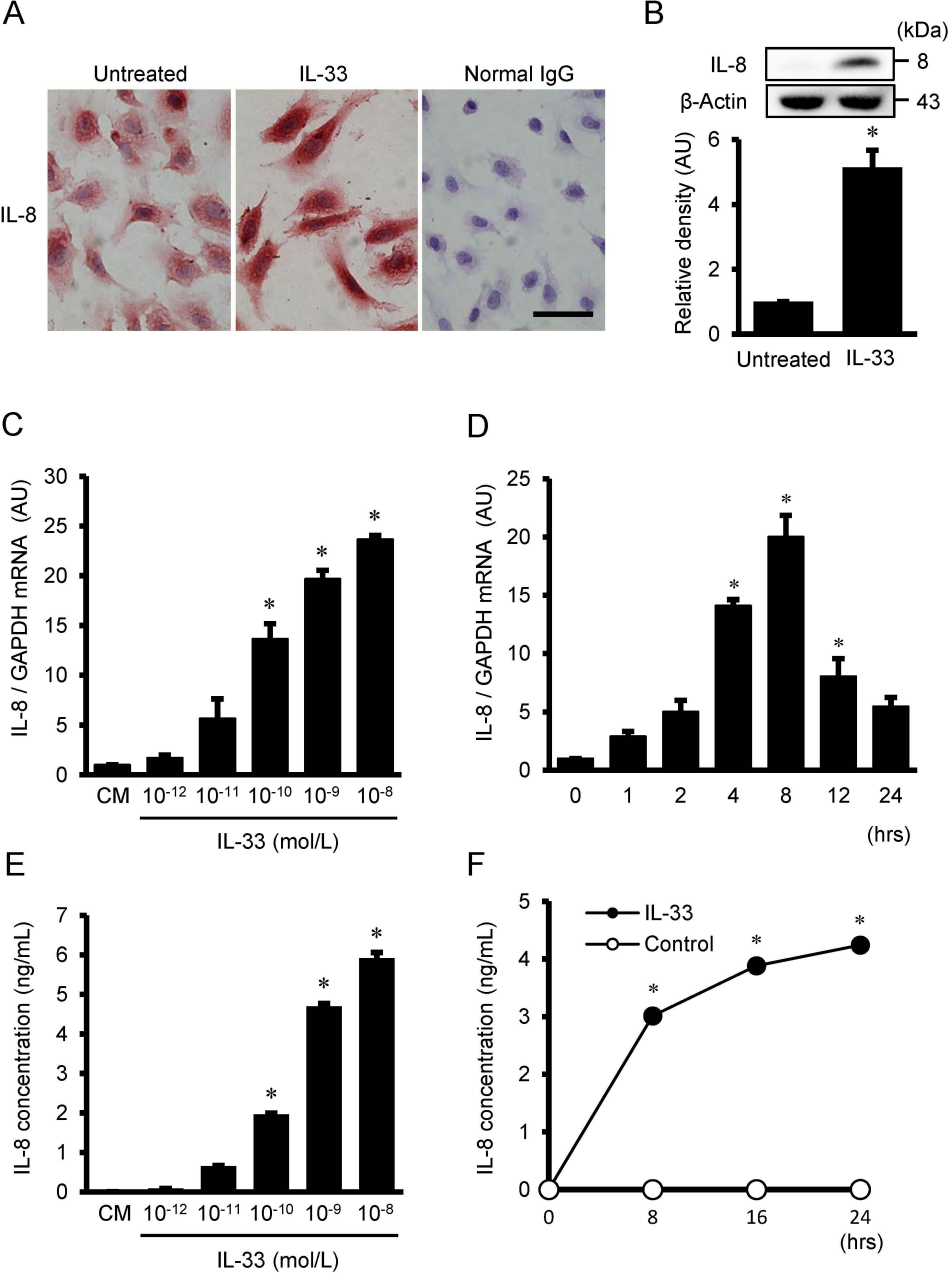
IL-33 stimulates protein expression, gene expression, and protein secretion of IL-8 in HUVECs. (A) Representative immunocytochemical staining showing localization of IL-8 in HUVECs with or without exposure to 10^−9^ mol/L IL-33 for 24 hrs. Intensities of immunoreactivity for IL-8 were increased in HUVECs treated with IL-33 compared with untreated cells. Normal mouse IgG served as a negative control. Original magnification; ×400. Scale bar = 50 μm. (B) Western immunoblot analysis showing that protein expression for IL-8 was increased in HUVECs treated with 10^−9^ mol/L IL-33 for 24 hrs. Bars represent densitometric analyses of each expression signal after normalization to expression of β-actin and relative to untreated control. Values are means ± SE of three independent experiments. *P <0.05 vs. untreated control. (C) IL-8 mRNA expression in HUVECs after treatment with the indicated concentrations of IL-33 for 4 hrs (n = 3), as evaluated by real time RT-PCR. (D) Time course of IL-8 mRNA after treatment with 10^−9^ mol/L IL-33 (n = 3), as evaluated by real time RT-PCR. Bars represent IL-8 mRNA after normalization to GAPDH mRNA and relative to conditioned medium (CM) in C and 0 hr in D. (E) IL-8 concentration in the supernatant after treatment with the indicated concentrations of IL-33 for 24 hrs (n = 6), as analyzed by ELISA. Bars represent IL-8 protein secretion per 10 ^5^ cells. (F) Time course of IL-8 concentration in the supernatant after treatment with 10^−9^ mol/L IL-33 (closed circles, n = 6), as analyzed by ELISA. Spontaneous secretion of IL-8 without IL-33 is less than the limit of detection levels (open circles, n = 6), as analyzed by ELISA. *P <0.05 vs. CM in C and E, and vs. 0 hr in D and F.

Immunocytochemical staining revealed that positive immunoreactivities for IL-8 were detected in the untreated HUVECs, and that the intensities of immunoreactivity for IL-8 were enhanced by the treatment with 10^−9^ mol/L of IL-33 for 24 hrs (Fig 1A). On the other hand, the cells treated with normal IgG, instead of primary antibody against IL-8, demonstrated little or no immunoreactivity. The presence of IL-8 in cultured HUVECs was further confirmed by Western immunoblot analysis. As shown in Fig 1B, a significant increase in IL-8 expression was observed after treatment with 10^−9^ mol/L of IL-33 for 24 hrs.

### IL-33-induced IL-8 gene expression and protein secretion in HUVECs

Real time RT-PCR revealed that HUVECs treated with IL-33 (10^−12^ to 10^−8^ mol/L) resulted in an increase in IL-8 mRNA in a dose-dependent manner with a statistical significance at 10^−10^ to 10^−8^ mol/L of IL-33 (Fig 1C). As shown in Fig 1D, IL-33 significantly increased IL-8 mRNA at 4 to 12 hours after IL-33 stimulation. ELISA showed that IL-33 increased IL-8 protein secretion from HUVECs in a dose-dependent manner with a significant increase at the doses over 10^−10^ mol/L of IL-33 (Fig 1E), and in a time-dependent manner with a significant increase at 8 to 24 hrs (Fig 1F). Spontaneous secretion of IL-8 into the culture medium of HUVECs was under the limit of detection levels by ELISA (Fig 1F).

### Comparison of the dose-dependent effects between IL-33 and IL-1β

**Fig 2:**
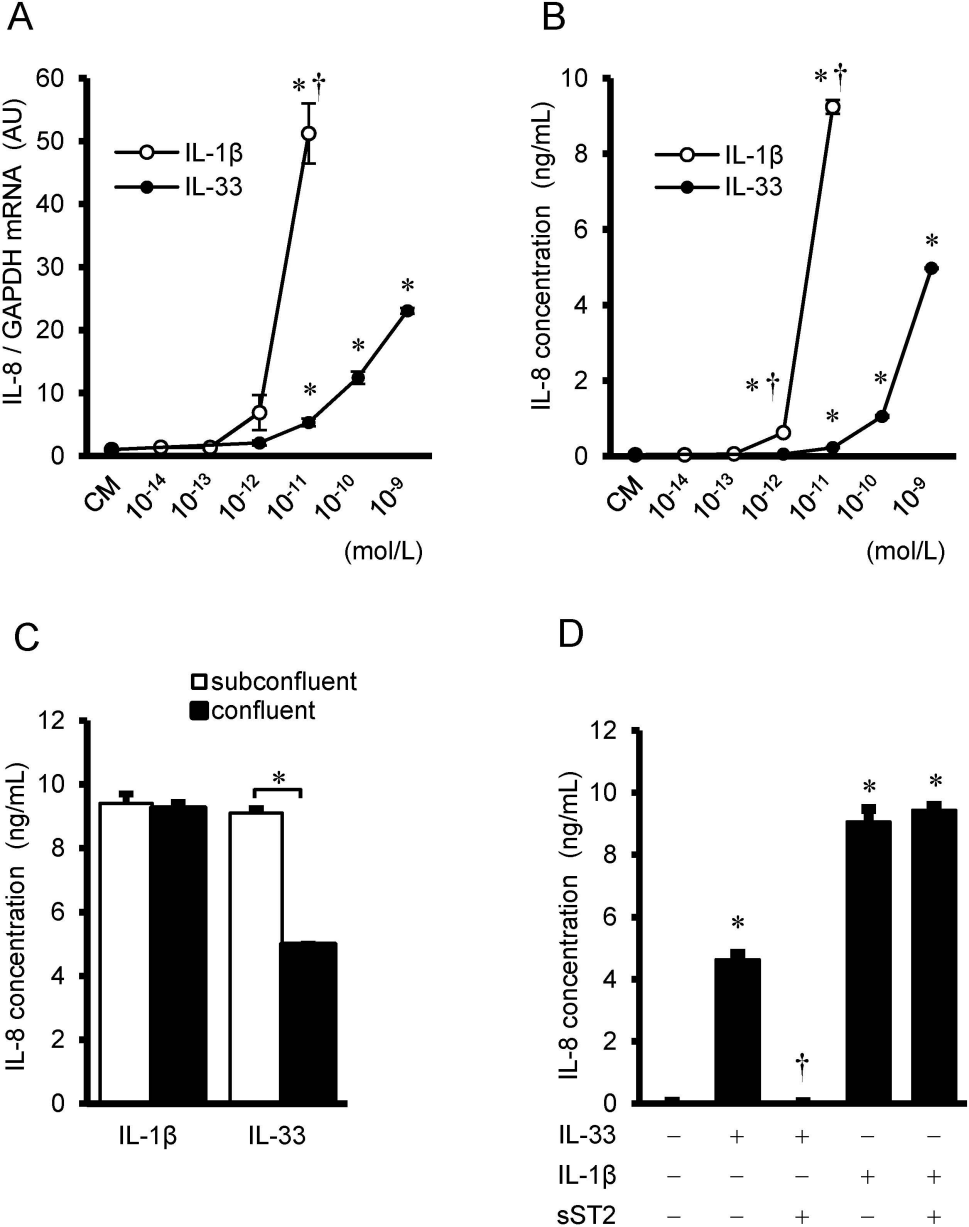
Comparison between IL-33 and IL-1β, and the effects of soluble ST2. (A) IL-8 mRNA expression in HUVECs after treatment with the indicated concentrations of IL-33 (closed circles) and IL-1β (open circles) for 4 hrs (n = 3), as evaluated by real time RT-PCR. Data represent IL-8 mRNA after normalization to GAPDH mRNA and relative to conditioned medium (CM). *P <0.05 vs. CM, †*P* <0.05 vs. IL-33 at respective concentrations. (B) IL-8 concentration in the supernatant after treatment with the indicated concentrations of IL-33 and IL-1βfor 24 hrs (n = 3), as analyzed by ELISA. Data represent IL-8 protein secretion per 10^5^ cells. *P <0.05 vs. CM, †*P* <0.05 vs. IL-33 at respective concentrations. (C) HUVECs were seeded at different densities (subconfluent: confluent = 1:3), cultured, and treated with IL-33 (10^−9^ mol/L) and IL-1β (10^−11^ mol/L) for 24 hrs (n = 3). IL-8 concentration per 10^5^ cells was measured by ELISA. *P <0.05 between confluent and subconfluent. (D) HUVECs were pre-incubated with soluble ST2 (sST2, 10^−7^ mol/L) for 2 hrs, followed by additional incubation with IL-33 (10^−9^ mol/L) or IL-1β (10^−11^ mol/L) for 24 hrs. IL-8 concentration per 10^5^ cells was measured by ELISA. *P <0.05 vs. untreated control, †*P* <0.05 vs. IL-33.

The effects of IL-33 on the gene expression and protein secretion of IL-8 were further compared with those of a prototype IL-1 family member, IL-1β. IL-1β increased the gene expression (Fig 2A) and protein secretion (Fig 2B) of IL-8 with more than two order higher magnitude of sensitivity compared with IL-33. To examine the responsiveness of IL-33 and IL-1β to the proliferating or non-proliferating quiescent cells, HUVECs were seeded at different densities, and the effects of IL-1β (10^−11^ mol/L) and IL-33 (10^−9^ mol/L) on the protein secretion of IL-8 were examined in the confluent or subconfluent conditions (S1 Fig). IL-33 (10^−9^ mol/L) increased IL-8 protein secretion more in the subconfluent conditions compared with the confluent conditions. On the other hand, increases in IL-8 protein secretion induced by IL-1β (at 10^−11^ mol/L) were not changed between the confluent and subconfluent conditions (Fig 2C). Excess amount of soluble ST2 (10^−7^ mol/L) blocked IL-33-induced IL-8 protein secretion, however IL-1β-induced IL-8 protein secretion was not affected by the addition of soluble ST2 (Fig 2D).

### IL-33-induced phosphorylation of JNK in HUVECs

**Fig 3:**
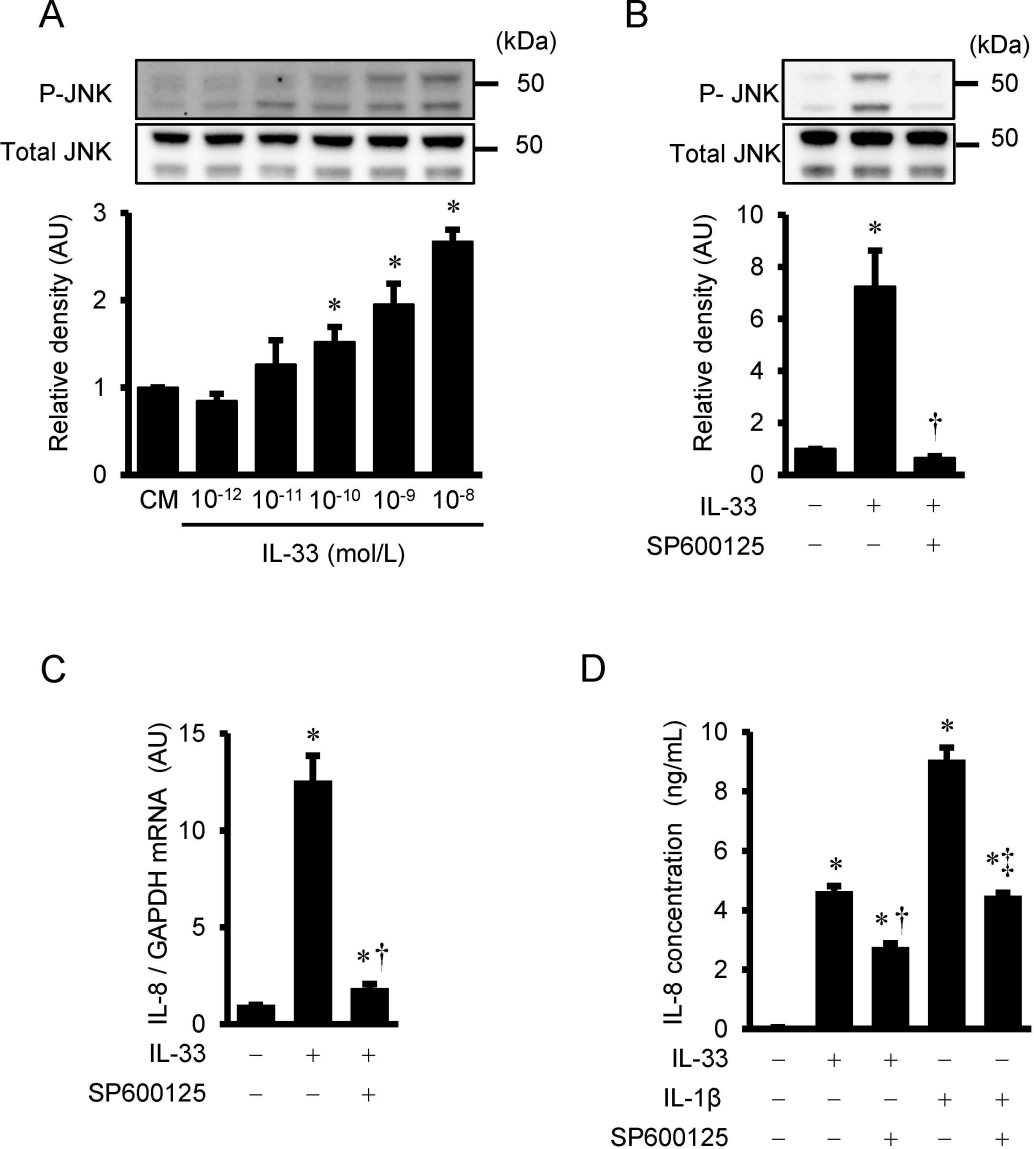
Effects of SP600125 on IL-33-induced JNK phosphorylation, IL-8 mRNA, and IL-8 protein secretion. (A) HUVECs were treated with IL-33 (10^−12^ to 10^−8^ mol/L) for 30 min. IL-33-induced phosphorylation of JNK as evaluated by Western immunoblot analysis. Bars represent results from densitometric analyses of each phosphorylation signal after normalization to total protein and relative to conditioned medium (CM). Blots are representative of three independent experiments. *P <0.05 vs. CM. (B) HUVECs were pretreated with SP600125 (JNK inhibitor, 30 μmol/L) for 2 hrs, and then incubated with IL-33 (10^−9^ mol/L) for 30 min. Bars represent results from densitometric analyses of each phosphorylation signal after normalization to total protein and relative to untreated control. Blots are representative of three independent experiments. *P <0.05 vs. untreated control. †*P* <0.05 vs. IL-33. (C and D) HUVECs were pre-incubated with SP600125 (30 μmol/L) for 2 hrs, followed by additional incubation with IL-33 (10^−9^ mol/L) for 4 hrs (C, IL-8 mRNA), and IL-33 (10^-9^mol/L) and IL-1β (10^-11^mol/L) for 24 hrs (D, IL-8 secretion). IL-8 mRNA and protein secretion were evaluated by real time RT-PCR (C, n = 3) and ELISA (D, n = 3), respectively. *P <0.05 vs. untreated control. †*P* <0.05 vs. IL-33. ‡*P* <0.05 vs. IL-1β.

IL-33 phosphorylates JNK in a dose-dependent manner with a statistical significance over 10^−10^ mol/L of IL-33 (Fig 3A). As shown in Fig 3B, IL-33-stimlated phosphorylation of JNK was completely suppressed by 30 μmol/L of the JNK inhibitor, SP600125.

### Effects of SP600125 on IL-33-induced IL-8 gene expression and protein secretion in HUVECs

To examine whether JNK pathway is involved in IL-33-induced IL-8 mRNA expression and protein secretion, HUVECs were pre-treated with SP600125 for 2 hours and then incubated with IL-33 (10^−9^ mol/L) for 4 hours to examine IL-8 mRNA expression, and for 24 hours to measure IL-8 protein secretion from HUVECs. Real time RT-PCR demonstrated that IL-33-induced increase in IL-8 mRNA expression was significantly attenuated by the pretreatment with SP600125 in HUVECs (Fig 3C). As shown in Fig 3D, an increase in IL-33-induced IL-8 protein secretion from HUVECs for 24 hours was also significantly suppressed by SP600125, however to a lesser degree as compared with IL-33-induced IL-8 mRNA expression. IL-8 protein secretion stimulated by IL-1β was also reduced by SP600125 (Fig 3D).

### IL-33-induced phosphorylation of c-Jun in HUVECs

**Fig 4:**
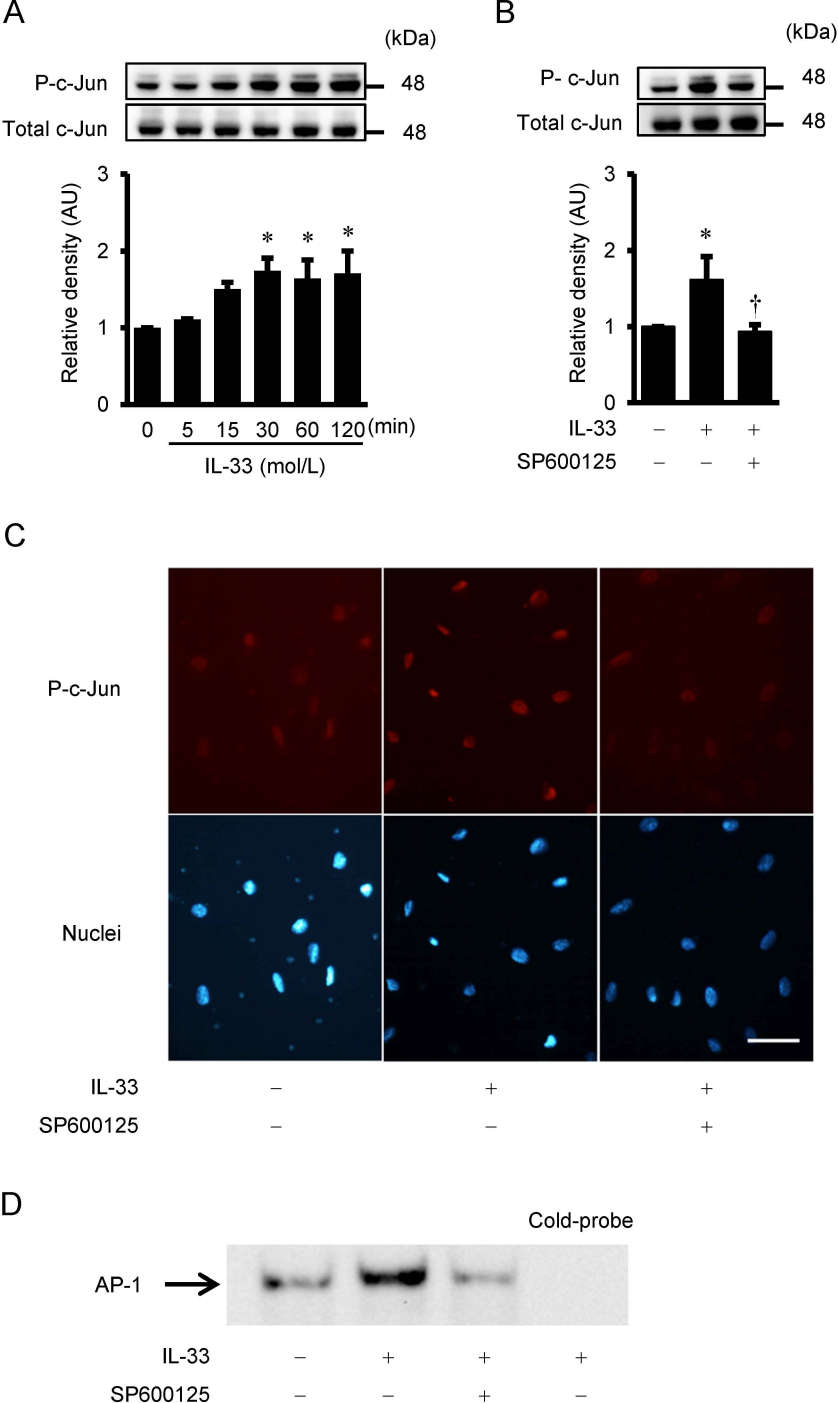
IL-33-induced c-Jun phosphorylation, immunofluorescence staining for phospho-c-Jun, and AP-1 DNA-binding activity. (A and B) IL-33-induced phosphorylation of c-Jun as evaluated by Western immunoblot analysis. (A) HUVECs were treated with IL-33 (10^−9^ mol/L) for the indicated time periods. (B) HUVECs were pretreated with SP600125 (30 μmol/L) for 2 hrs, and then incubated with IL-33 (10^−9^ mol/L) for 60 min. Bars represent results from densitometric analyses of each phosphorylation signal after normalization to total protein and relative to 0 min in A or relative to untreated control in B. Blots are representative of three independent experiments. *P <0.05 vs. 0 min in A or vs. untreated control in B. †*P* <0.05 vs. IL-33 in B. (C) HUVECs were pre-incubated with SP600125 (30 μmol/L) for 2 hrs and then incubated with IL-33 (10^−9^ mol/L) for 60 min. Red staining indicates the specific Alexa staining for phospho-c-Jun, and blue staining indicates the nuclei (Hoechst 33342). Original magnification; ×400. Scale bar = 50 μm. (D) IL-33-increased AP-1 binding activity, as measured by electrophoretic mobility shift assay (EMSA). HUVECs were pretreated with SP600125 (30 μmol/L) for 2 hrs and then incubated with IL-33 (10^−9^ mol/L) for 60 min. Unlabeled probe (cold-probe) was used for competition to verify that the bands were AP-1 specific.

To determine the downstream signaling pathway of JNK, phosphorylation of c-Jun was assessed by Western immunoblot analysis. IL-33 stimulated phosphorylation of c-Jun in a time-dependent manner with a statistical significance over 30 min of treatment with 10^−9^ mol/L of IL-33 (Fig 4A). IL-33-stimulated c-Jun phosphorylation was reduced by the pre-treatment with the JNK inhibitor, SP600125 (Fig 4B).

### Immunofluorescence staining

Immunofluorescence signal of phospho-c-Jun was significantly located in the nuclei of HUVECs after treatment with IL-33 for 60 min compared with untreated control (Fig 4C). The phospho-c-Jun activation by IL-33 was attenuated by the JNK inhibitor, SP600125.

### IL-33-induced activation of AP-1 in HUVECs

EMSA revealed that IL-33 increased the amount of DNA-protein complex with the AP-1 probe, and this complex was suppressed by SP600125 (Fig 4D). Addition of an excess amount of cold probe abolished the IL-33-induced increase in the DNA-protein complex.

### Effects of c-Jun siRNA transfection on the secretion of IL-8 from HUVECs

**Fig 5:**
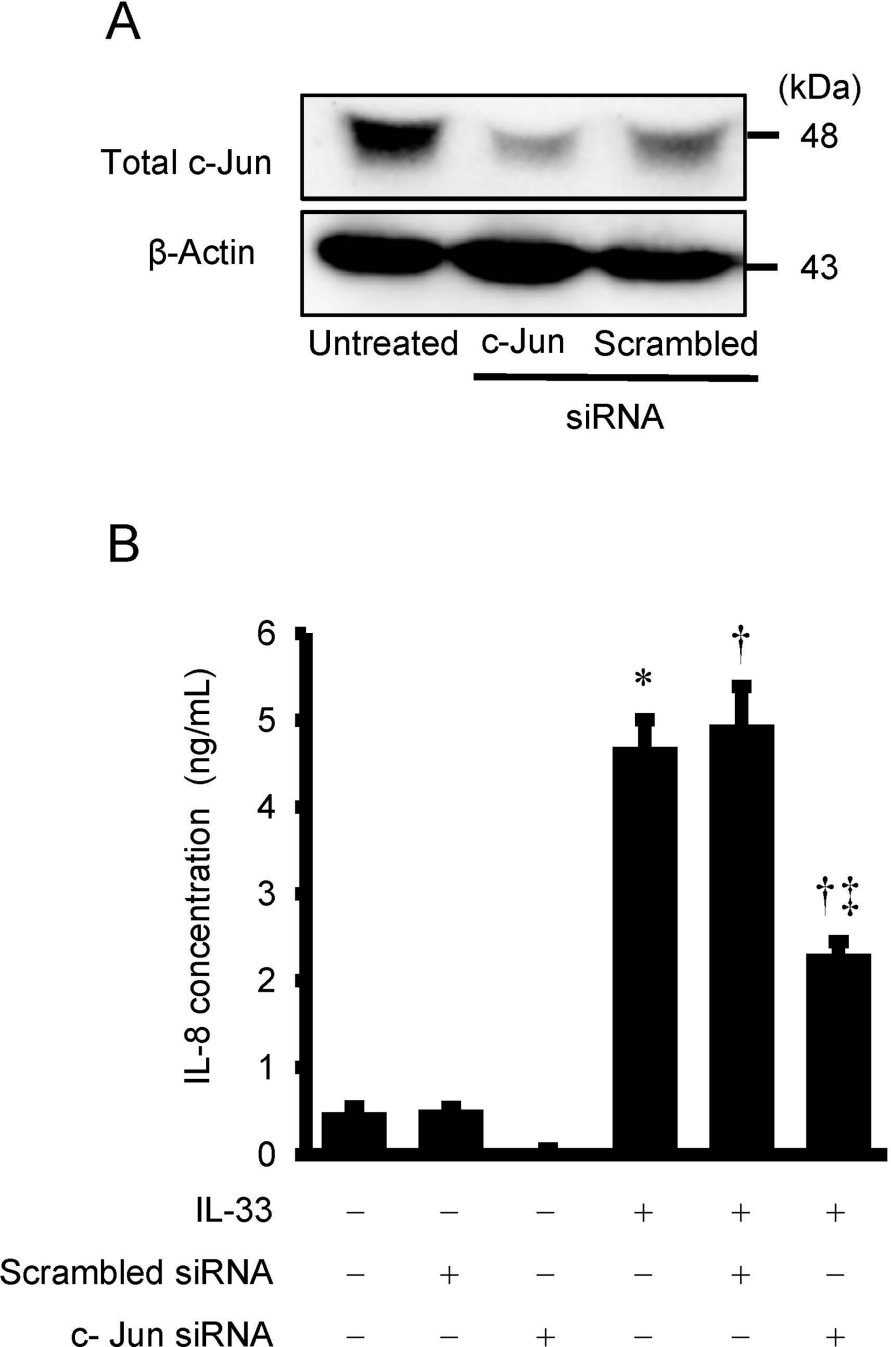
Effects of c-Jun siRNA on IL-33-induced IL-8 secretion from HUVECs. Antibody-free HUVECs were transfected with control scrambled siRNA or c-Jun siRNA (100 nmol/L) for 48 hrs and then stimulated with or without IL-33 (10^−9^ mol/L) for 24hrs. (A) Cell lysates were evaluated for knock down of c-Jun by Western immunoblot analysis. β-Actin was used for loading control. (B) IL-8 concentration in the supernatant as measured by ELISA (n = 6). Bars represent IL-8 protein secretion per 10 ^5^ cells. *P <0.05 vs. untreated control. †*P* <0.05 vs. scrambled siRNA, ‡*P* <0.05 vs. scrambled siRNA plus IL-33.

To further confirm the role of c-Jun in IL-33-induced IL-8 secretion, we transfected antibody-free HUVECs with c-Jun siRNA. Efficacy of transfection of siRNA was shown in Fig 5A. Transfection of c-Jun siRNA for 48 hrs reduced c-Jun expression by 49% compared with control scrambled siRNA transfection (Fig 5A). Transfection of c-Jun siRNA significantly attenuated an increase in IL-8 secretion stimulated by IL-33 (Fig 5B).

## Discussion

The present study has demonstrated that IL-33, a member of the IL-1 cytokine family, induces the expression of IL-8, a member of the CXC-chemokine family in HUVECs. This study revealed the following findings. 1) Immunocytochemical examination and Western immunoblot analysis demonstrated that IL-33 increased the IL-8 protein expression in HUVECs. 2) Real-time PCR and ELISA showed that IL-33 increased IL-8 gene expression and protein secretion in a dose- and time-dependent manner in HUVECs, and the dose-dependent responses to IL-33 were weaker than those to IL-1β. 3) IL-33 preferentially stimulated subconfluent cells, resulting in the increased IL-8 protein secretion to a higher level compared with confluent cells. 4) IL-33 stimulated phosphorylation of JNK and c-Jun in HUVECs. 5) IL-33 induced translocation of transcription factor AP-1 to the nucleus in HUVECs. 6) EMSA revealed that IL-33 increased AP-1 DNA-binding activity in HUVECs. 7) Transfection of c-Jun small interfering RNA reduced the IL-33-induced increase in IL-8 secretion from HUVECs. 7) SP600125, a pharmacological inhibitor of JNK, inhibited IL-33-induced IL-8 gene and protein expression, phosphorylations of JNK and c-Jun, translocation of AP-1 to the nucleus, and AP-1 DNA-binding activity in HUVECs.

IL-33 and its receptor ST2 are associated with a variety of inflammatory diseases, such as asthma, rheumatoid arthritis, atopic dermatitis, inflammatory bowel disease, and cardiovascular disease including atherosclerosis [13]. IL-33 induces various proinflammatory and atherogenic factors, including inflammatory cytokines, adhesion molecules, chemokines, coagulation factors, and proteolytic factors [18, 22, 30–34]. In our previous study, we have demonstrated that IL-33 increases expression of GRO-α, a member of CXC-chemokine family, in HUVECs [20]. IL-8, another member of CXC-chemokine family, is a major chemoattractant and angiogenic factor [35], and until now, a few studies have documented IL-33-induced IL-8 expression in endothelial cells and epithelial cells [18, 22–24]. Aoki et al. demonstrated that IL-33 increased secretions of IL-6 and IL-8 from HUVECs in a dose-dependent manner [24]. Demyanets et al. also reported that IL-33 up-regulated IL-8 protein expression in explanted atherosclerotic plaque tissue [18]. Yagami et al. [22] and Lin et al. [23] demonstrated IL-33-induced IL-8 expression in other type of cells, such as human lung tissue cells and human corneal epithelial cells, respectively. The present study has confirmed these previous studies, and has demonstrated that IL-33 induces IL-8 gene and protein expression in human vascular endothelial cells.

In the present study, we have compared the effects of IL-33 on the gene expression and protein secretion of IL-8 with those of IL-1β, and found that the responses to IL-33 were much less compared with those to IL-1β. Interestingly, the levels of confluence influenced the responsiveness of HUVECs to IL-33. IL-33 preferentially stimulated the proliferating cells, and increased IL-8 protein secretion to a higher level compared with the confluent quiescent cells. These findings were in agreement with the previous studies that IL-33 preferentially stimulates proliferating non-quiescent cells [17]. Because the membrane-bound ST2L, a receptor of IL-33, is rich in the proliferating cells [17, 24], and is decreased in the quiescent cells, the effects of IL-33 on IL-8 protein secretion may be considered stronger in the proliferating cells, depending on the numbers of ST2L. On the other hand, there were no significant differences in the IL-1β stimulated IL-8 protein secretion between the confluent conditions and subconfluent conditions, suggesting that the levels of confluence has no influences on the responsiveness of HUVECs to IL-1β.

IL-8 is a potent modulator of atherosclerosis progression. Previous studies have shown that IL-8 expression is present and increased in human atherosclerotic lesions [4, 5, 36, 37]. In addition, IL-8 was a powerful trigger for firm adhesion of monocytes to vascular endothelium [38], which is a key event involved in the initiation and progression of atherosclerosis. In the present study, we have demonstrated that IL-33 increases IL-8 expression in cultured vascular endothelial cells. Although Demyanets et al. detected IL-33 and ST2 on both gene and protein levels in human carotid atherosclerotic plaques [18], the role of IL-33 in the pathophysiology of atherosclerosis has not been fully understood yet. IL-33 would be associated with the process of atherogenesis by increasing IL-8 production via the IL-33/ST2 system, and therefore, further studies are needed to determine whether IL-33-induced IL-8 is associated with the pathophysiology of atherosclerosis.

JNK is activated by various cytokines and environmental stresses, and it subsequently regulates phosphorylation of c-Jun and AP-1 transcription activity [39]. Accumulating evidence has demonstrated that JNK plays an important role in the biological actions of IL-33 in the normal cells and the cancer cells [40–42]. Ashlin et al. reported that IL-33 mediated activation of c-Jun and JNK in human macrophages, and RNA interference assays revealed that c-Jun and JNK were involved in transducing the reduction of a disintegrin and metalloproteinase with thrombospondin motifs (ADAMTS)-1 and -4 expressions by IL-33 [41]. Ye et al. demonstrated that IL-33 increased the activation of JNK, and inhibition of JNK by SP600125 significantly blocked the protective effects of IL-33 in gastric cancer cells [42]. As for the signal transduction pathways involved in IL-33-induced IL-8 expression, a few studies described the involvement of p38 MAPK and ERK1/2 signaling pathways. IL-33-induced production of IL-8 was mediated by p38 MAPK in human umbilical cord blood-derived mast cells [43], or by ERK1/2 signaling pathway both in human nasal epithelial cells[40] and in retinal pigment epithelial cells [44]. Yagami et al. reported that IL-33 induced activation of both ERK1/2 and p38 MAPK in microvascular endothelial cells, and only ERK1/2 in bronchial epithelial cells. They also showed that p38 MAPK was required for the IL-33-mediated IL-8 production in microvascular endothelial cells, while ERK1/2 was needed for that in bronchial epithelial cells [22]. However, no studies have elucidated the importance of the JNK signaling pathway involved in the IL-33-induced IL-8 activation in the literature. In the present study, IL-33 stimulated phosphorylation of JNK, and JNK inhibitor SP600125 reduced IL-33-mediated increases in IL-8 gene expression and secretion in HUVECs. Further, IL-33 activated phosphorylation of c-Jun and enhanced AP-1 DNA binding activity in HUVECs, which was also suppressed by SP600125. Furthermore, IL-33-induced increases in IL-8 secretion were suppressed by c-Jun siRNA in HUVECs. These findings have indicated that IL-33-induces IL-8 expression through the JNK/c-Jun/AP-1 pathway in HUVECs. In support of our results, IL-1, an original member of IL-1 cytokine family, induces IL-8 production through the activation of JNK in human airway smooth muscle cells [45], and in epidermal cancer cells [46]. Further studies are required to explore the role of the JNK pathway in the activation of IL-33/ST2 system in various types of cells.

In conclusion, the present study has demonstrated that IL-33 induces IL-8 gene and protein expression through the activation of JNK/c-Jun/AP-1 pathway in HUVECs. We speculate that the increase in local and circulating IL-33 levels in patients with atherosclerotic and/or inflammatory diseases would stimulate the vascular endothelial cells to enhance IL-8 production via JNK-c-Jun-AP-1 pathway, contributing to the development of inflammatory disorders including atherosclerosis. The present study provides a new insight into the role of IL-33-induced IL-8 expression in the pathophysiology of atherosclerosis and vascular inflammation.

## Supporting information

S1 Fig Confluent and subconfluent conditions. HUVECs were cultivated at different densities and stimulated with IL-33 and IL-1β for 24 hrs.
(TIF)
