# Supplementary figures and images for: Interleukin-33 induces interleukin-8 expression via JNK/c-Jun/AP-1 pathway in human umbilical vein endothelial cells
Source: PLoS One. 2018 Jan 26;13(1):e0191659. doi: 10.1371/journal.pone.0191659 (PMC5786299; doi:10.1371/journal.pone.0191659)

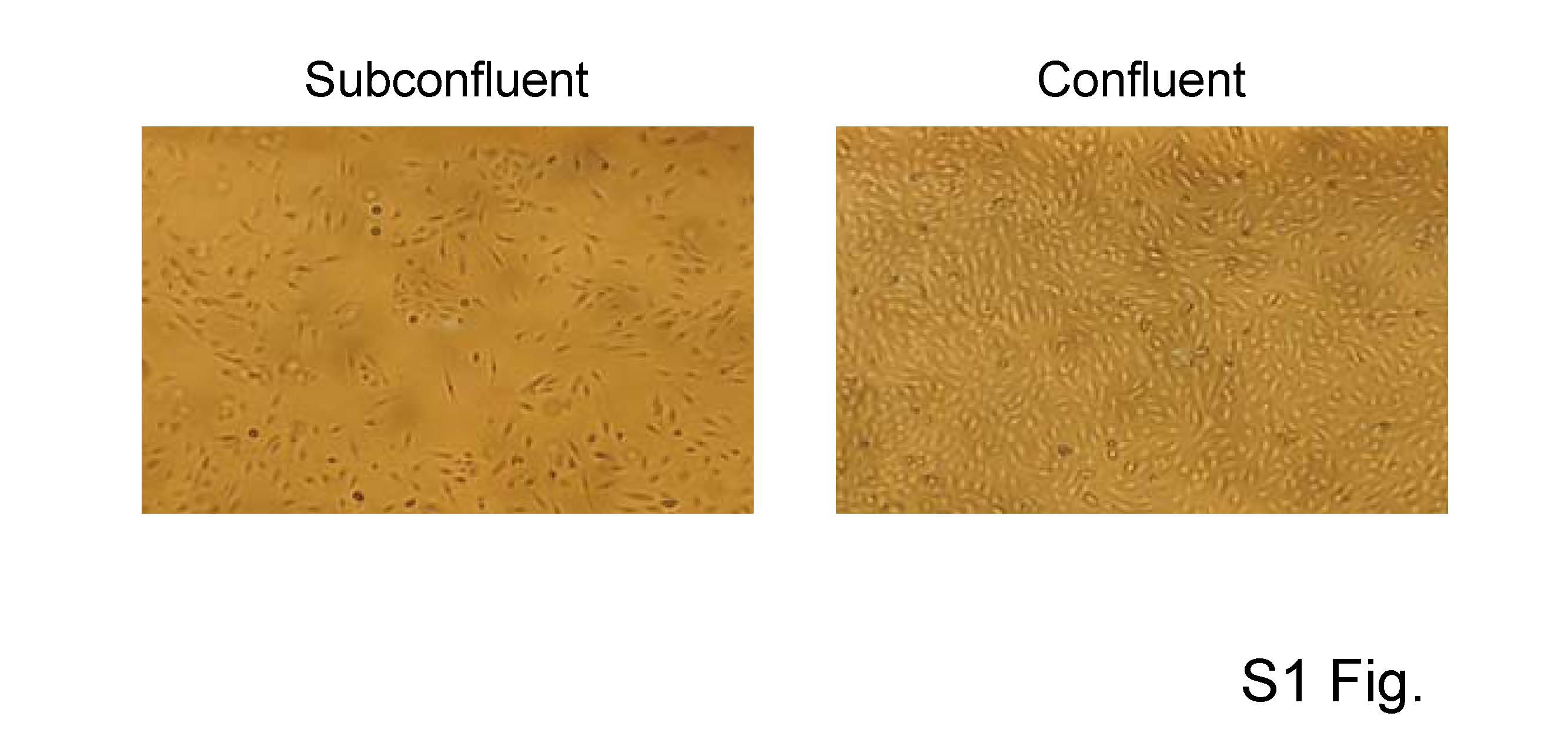

Supplement: S1 Fig — HUVECs were cultivated at different densities and stimulated with IL-33 and IL-1β for 24 hrs. (TIF) [file pone.0191659.s001.tif]
